# Specific Metabolites in a *Phaeodactylum tricornutum* Strain Isolated from Western Norwegian Fjord Water

**DOI:** 10.3390/md14010009

**Published:** 2015-12-30

**Authors:** Siv Kristin Prestegard, Svein Rune Erga, Pia Steinrücken, Svein Are Mjøs, Gjert Knutsen, Jens Rohloff

**Affiliations:** 1 Bergen Marine Biobank, Department of Biology, University of Bergen, Thormøhlensgate 53A/B, N-5020 Bergen, Norway; 2 Applied Biotechnology, Uni Research Environment, Thormøhlensgate 49B, N-5006 Bergen, Norway; 3 Department of Biology, University of Bergen, Thormøhlensgate 53AB, N-5020 Bergen, Norway; svein.erga@uib.no (S.R.E.); pia.steinrucken@uib.no (P.S.); gjert.knutsen@uib.no (G.K.); 4 Department of Chemistry, University of Bergen, Allégaten 42, N-5020 Bergen, Norway; svein.mjos@uib.no; 5 Department of Biology, Norwegian University of Science and Technology, N-7491 Trondheim, Norway; jens.rohloff@ntnu.no

**Keywords:** *Phaeodactylum tricornutum*, two-dimensional gel electrophoresis (2DGE), volatile organic compounds (VOCs), hormosirene, ectocarpene, fatty acids, eicosapentaenoic acid (EPA)

## Abstract

We have searched for special characteristics in growth, protein expression, fatty acids and volatile organic compounds (VOCs) in a local *Phaeodactylum tricornutum* Bohlin strain (Bergen Marine Biobank), by comparing it with a common accession strain (CCAP). Differences in growth and expressed proteins were detected between the BMB strain and the CCAP strain, and the BMB strain reached the highest cell densities under the given growth conditions. Fatty acid (FA) analyses showed highest relative eicosapentaenoic acid (EPA) levels in the exponential phase (25.73% and 28.31%), and highest levels of palmitoleic acid (16:1 *n*-7) in the stationary phase (46.36% and 43.66%) in the BMB and CCAP strain, respectively. The most striking finding of the VOCs analyses was the relatively high levels of ectocarpene, 6-((1*E*)-butenyl)-1,4-cycloheptadiene, hormosirene, and desmarestene and structurally related compounds, which were exclusively detected in the BMB strain. Many of the VOCs detected in the CCAP and, in particular, in the BMB strain have been reported as antimicrobial agents. We suggest that the array of pheromones and antimicrobial substances could be part of an allelopathic strategy of the BMB strain, dominated by oval cells, thus reflecting the benthic life stage of this morphological form. These findings show the potential for bioactive metabolites in the BMB strain.

## 1. Introduction

The diatoms represent a large and extraordinary ecologically flexible group of unicellular eukaryotic microalgae. They have acquired genes from an eukaryotic heterotroph and a red algae through secondary endosymbiosis [1] and later through lateral gene transfers from bacteria [2]. The complex genomes of diatoms, together with their ability for sexual reproduction [3], give this genetically highly diverse group many interesting metabolic properties.

Except for the synthesis of primary metabolites like pigments and certain proteins, it is currently impossible to foresee how diatoms will respond metabolically to different sets of growth or stress conditions. The biogenesis of secondary metabolites in diatoms and microalgae in general is unpredictable and needs to be investigated thoroughly. The capability of cells to produce bioactive compounds must therefore be examined empirically, and the increasing knowledge of diatom metabolites was excellently reviewed by Stonik, V. and I. Stonik [4]. The sequenced genome of *P. tricornutum* in 2008 [2] has provided new insight into the molecular foundation of adaption and survival mechanisms to highly changing environments [5,6]. It has been shown that diatoms excrete many of their bioactive compounds, which can have a variety of influences on other species in the plankton community [7,8]. Recently, metabolic fingerprinting of metabolites that had been excreted into the surrounding medium from the diatoms *Skeletonema marinoi* and *Thalassiosira pseudonana* revealed growth stage dependence [9]. Thus, volatile metabolites originating from exudates from living cells, can also give important information concerning physiological status, and several volatile compounds have been reported to be derivatives from degradation of fatty acids [10,11].

The polymorphic diatom *Phaeodactylum tricornutum* Bohlin is recognized as a model organism for studies on diatom physiology and genetics. It is also used on an industrial level for aquaculture feed because of its high content of polyunsaturated fatty acids (PUFAs), especially eicosapentaenoic acid (EPA), which is of high value as a food additive to human and animals [12,13].

*Phaeodactylum tricornutum* can be found in both brackish and marine waters worldwide [14]. Environmental conditions have been reported to induce the transformation between the different morphological cell forms; oval, fusiform, triradiate and cruciform [15,16,17,18]. Oval cells of *P. tricornutum* are considered to represent a benthic life stage since they are most often observed when grown on agar or on the surface of culture containers [19,20].

From a local strain of *P. tricornutum,* isolated from fjord water in Western Norway [21], more than 95% of the cells were reported to be oval when grown in liquid bubbled cultures [22]. Water extracts from this strain strongly inhibited blood platelet activation and induced cell death in leukaemia cells. Part of the activity was shown to originate from the high levels of the nucleoside adenosine, while the rest of the activity was shown to originate from other unidentified compounds [21]. The highly interesting metabolic and morphological properties of this isolate motivated us to search for additional special characteristics in growth, protein expression, fatty acids and volatile organic compounds. Fatty acids are also interesting in terms of commercial applications, and they have been reported to be precursors for many volatile metabolites [10,11]. Volatile organic compounds (VOC) are part of the metabolome in diatoms and show a high structural variety [10]. Therefore, we wanted to shed light on the VOC fraction in order to reveal characteristic metabolites.

To improve the evaluation of our results, we compared it with a common CCAP strain (CCAP 1052/1A from the Culture Collection of Algae and Protozoa in Oban, UK). Here, we present novel data which make us believe that our local strain has potential for new bioactive metabolites, among which the VOC should be specially emphasized. These compounds comprise both pheromones and antimicrobial substances, which are of great ecological and biomedical importance.

## 2. Results

### 2.1. Different Growth and Protein Expression of BMB and CCAP Strains

**Figure 1:**
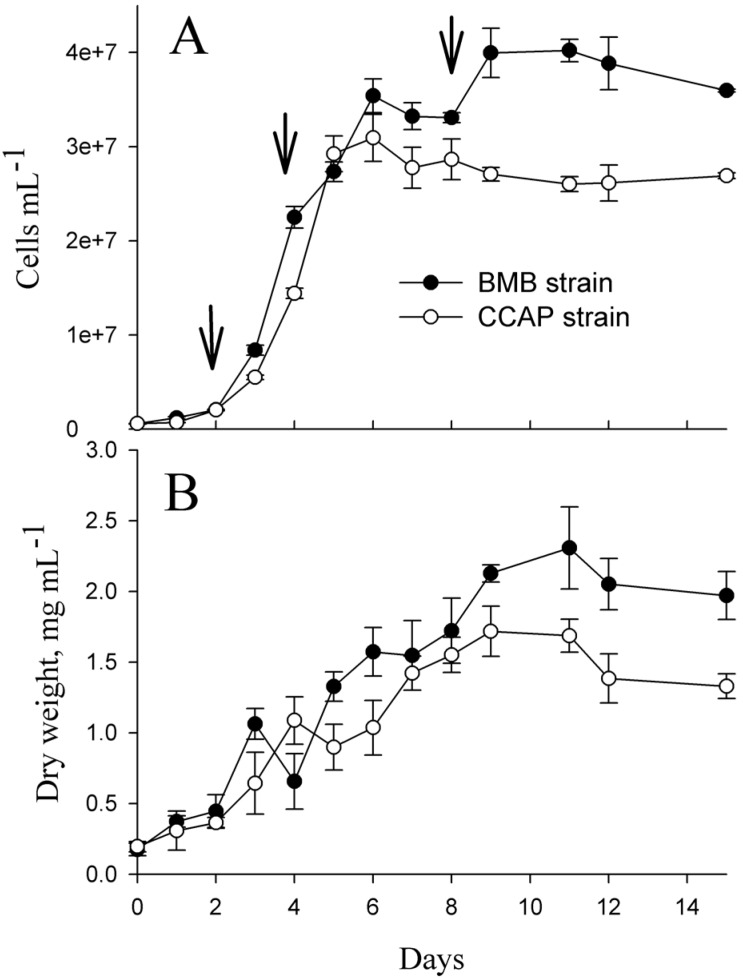
Growth of the BMB and CCAP strains in batch cultures. Cells were grown in artificial seawater with the addition of Walne’s medium, 27 PSU at 20 °C and 249 µmol photons m^−2^·s^−1^. (**A**): Cells·mL^−1^. Data in Figure 1A are modified from [22]. Arrows shows the time-points for sampling of total soluble proteins. (**B**): Dry weight mL^−1^ algae culture. The data in Figure 1A,B are from the same experiments.

Growth curves for the BMB (BMB-E-0007) and CCAP (CCAP 1052/1A) strains are presented in Figure 1A,B. From cell counts, the growth rates corresponding to the sampling day for soluble proteins were determined for both strains. The average growth rate was 0.58 ± 0.10 day^−1^ for the BMB strain and 1.07 ± 0.03 day^−1^ for CCAP from day one to day two. From day three to four, the average growth rate for the BMB strain was 1.01 ± 0.06 day^−1^ and 0.98 ± 0.03 day^−1^ for the CCAP strain. The CCAP cultures reached their maximal average cell densities of 3.1 × 10^7^ cells·mL^−1^ at day six, and then the cell numbers started to decrease slowly. The BMB strain cultures reached their maximal average cell densities of 4 × 10^7^ cells·mL^−1^ at day eleven, and at this time the cultures also obtained the maximal dry weights (Figure 1B). The CCAP strain reached it maximal dry weight at day nine.

**Figure 2:**
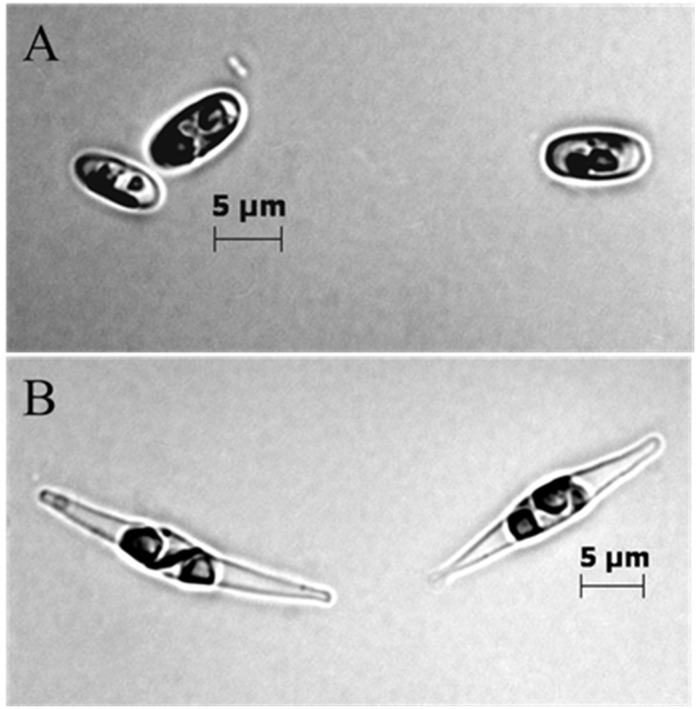
Microscope photos of BMB strain (**A**) and CCAP (**B**). A Zeiss Axio Imager Z1 microscope (Carl Zeiss) was used to photograph representative cells from the two strains (100× magnification with immersion oil, Bright Field (**A**) and Differential interference contrast (**B**)).

The BMB strain was dominated by oval cells (more than 95%), and the CCAP strain was dominated by cells with fusiform morphology (Figure 2A,B). Triradiate cells could occur in the CCAP strain, but it was never observed in the BMB strain.

**Figure 3:**
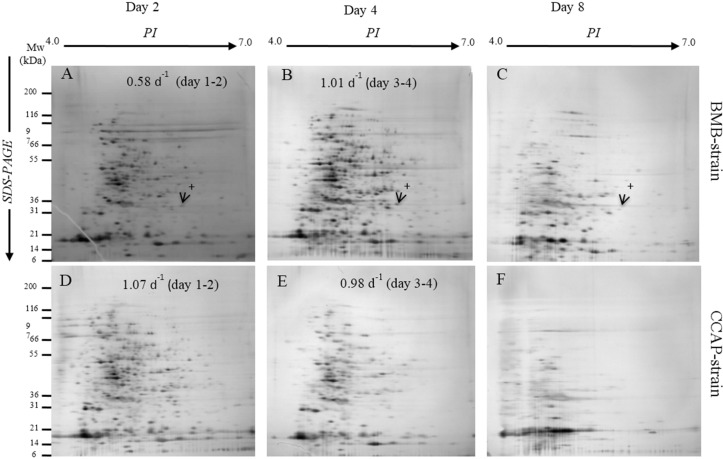
Representatives of protein profiles on 2DGE from BMB strain; (**A**): day 2; (**B**): day 4; (**C**): day 8 and of the CCAP strain; (**D**): day 2; (**E**): day 4; (**F**): day 8. Arrows with cross sign show that protein was present only in BMB strain.

Total soluble proteins separated by 2DGE from the different growth phases from the BMB and CCAP strains are presented in Figure 3A–F. The protein profiles differed according to growth phases and strain. Total soluble protein and number of resolved spots are presented in Table 1. At day two, more protein spots were resolved from the CCAP strain (372 ± 41) than from the BMB strain (197 ± 11), while at day four the number increased for the BMB strain (289 ± 18) and were reduced for the CCAP strain (242 ± 39).

Furthermore at day eight, in the stationary growth phase, especially the CCAP strain had strongly reduced resolved protein profiles on the 2DGE gels (Figure 3F). The protein profiles from the BMB strain in the stationary growth phase also revealed reduced number of proteins, but to a lesser extent than in the CCAP profiles (Figure 3C). Differences in the total soluble protein from samples preparations in the CCAP strain (3.12 ± 0.30) at day 2 compared to the BMB strain (2.56 ± 0.45) were not significant. Furthermore, the levels decreased by 24 times until day eight in the CCAP strain. The total soluble protein from the BMB strain also decreased towards day eight, but only by 10 times (Table 1). In Figure 3A–C), an example of a protein that appears to be specific for the BMB strain with an apparent molecular mass (Mw) of 33 kDa and *p*I of about 6.2 is marked. The 33 kDa protein was absent from the 2DGE gels separating proteins from the CCAP strain at any growth stage.

**Table 1:** Total soluble proteins cell^−1^ for samples from BMB and CCAP strain of *Phaeodactylum tricornutum* for different time points during growth in batch cultures (*n* = 3). Numbers of resolved spots using 2DGE are listed for the different samples (*n* = 3).

Strain	Day	Total Soluble Protein, pg/Cell	Resolved Protein Spots by 2DGE
**BMB**	2	2.56 ± 0.45	197 ± 11
	4	0.57 ± 0.06	289 ± 18
	8	0.25 ± 0.04	198 ± 19
**CCAP**	2	3.12 ± 0.30	372 ± 41
	4	1.10 ± 0.10	242 ± 39
	8	0.13 ± 0.02	90 ± 6

### 2.2. Fatty Acid Content and Composition of BMB and CCAP Strains

**Figure 4:**
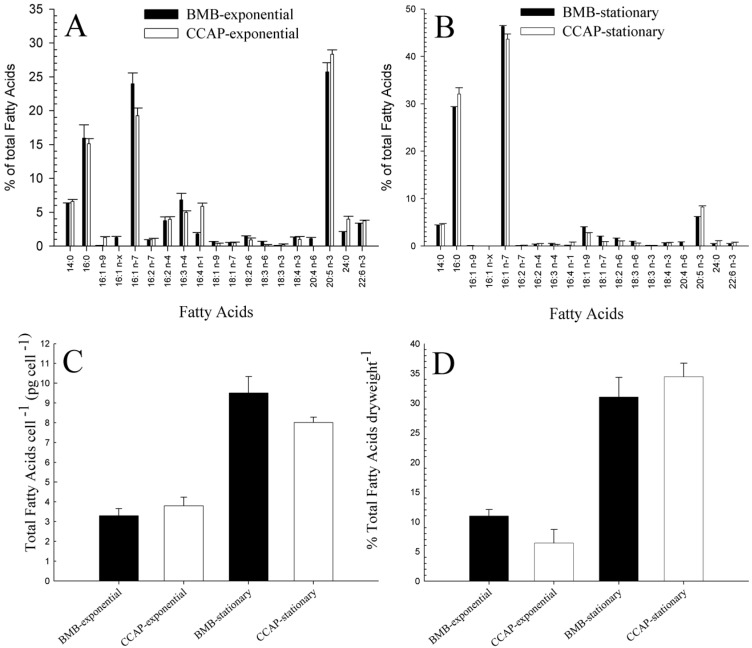
Fatty acid (FA) profiles given as % of total FA of BMB and CCAP strain in exponential phase (**A**) and in stationary phase (**B**). Total FA per cell in BMB and CCAP strain in exponential and stationary phase (**C**) and % of total FA per dry weight (**D**).

Samples taken from exponential and stationary phases of the BMB and CCAP strain were analysed for total fatty acid (FA) content and the relative composition of different FAs. In Figure 4A,B, 19 of the detected FA with relatively levels higher than 1% are shown. The two strains possess a similar FA composition in both growth phases. In the exponential phase (Figure 4A), eicosapentaenoic acid (EPA) (20:5 *n*-3) was the most dominant FA, with 25.73% ± 1.35% for the BMB strain and 28.31% ± 0.68% for the CCAP strain. The average cell specific content of EPA is 0.85 and 1.08 pg·cell^−1^ for exponentially growing BMB and CCAP strains, respectively, and 0.59 and 0.66 pg·cell^−1^, respectively, for stationary cells (see Figure 4A–C). In the stationary phase (Figure 4B), palmitoleic acid (16:1 *n*-7) became the predominant FA with 46.36% ± 0.16% of the total FA in the BMB strain and 43.66% ± 1.05% in the CCAP strain. Also, the levels of the saturated palmitic acid (16:0) had increased to 29.3% and 32.1% in the BMS and CCAP strain, respectively. EPA levels were reduced to 6.17% ± 0.06% for the BMB strain and 8.26% ± 0.21% for the CCAP strain in the stationary phase.

In the exponential growth phase, a higher relative fraction of the fatty acids consisted of long chain fatty acids and polyunsaturated FAs, whereas in the stationary phase, a higher fraction of the shorter fatty acids (C16 and 16:1 *n*-7) was present. The total amount of fatty acids per cell more than doubled from exponential phase to stationary phase for both strains (Figure 4C). In the stationary phase, the BMB strain had a slightly higher total FA per cell (9.51 pg ± 0.82) than the CCAP strain (8.02 pg ± 0.26). In the exponential phase, 10.98% ± 1.1% of the dry biomass in the BMB strain was FA, and in the stationary phase it had increased to 31.01% ± 3.31%. In the CCAP strain, 6.4% ± 2.27% of the dry algae biomass was fatty acids in the exponential phase and 34.44% ± 2.26% in the stationary phase (Figure 4D).

### 2.3. Differences in Volatile Organic Compounds (VOCs) from BMB and CCAP Strains

VOCs from early stationary cultures of BMB and CCAP strain of *P. tricornutum* were collected and analysed by headspace SPME–GC-MS. A broad range of 61 structurally different VOCs were tentatively identified, comprising aliphatics (6 alkanes, 4 aldehydes, 1 ketone, 4 acids, 2 esters), sulphides (3), terpenes (3 monoterpenes, 1 sesquiterpene, 1 triterpene), aromatics (3), furans (1), quinones (1), carotenoid-derived volatiles (6), and alicyclic olefins (18). Furthermore, 7 non-identified peaks were structurally assigned as aliphatic hydrocarbons (4) and aromatics (3) (Table 2). In general, a distinct higher number of VOCs could be isolated from the BMB strain (55) compared to the CCAP strain of *P. tricornutum* (36). The most striking observation in the BMB strain was the abundance of the algal pheromones ectocarpene, 6-((1*E*)-butenyl)-1,4-cycloheptadiene, hormosirene and desmarestene. Further 14 non-identified pheromonal volatiles were structurally annotated based on their characteristic and similar MS fragmentation patterns and the occurrence of the molecular ions of identified pheromones with (M+) *m/z* = 148 (compounds No. 22–24) and (M+) *m/z* = 146 (compound No. 30) (Table 2).

**Figure 5:**
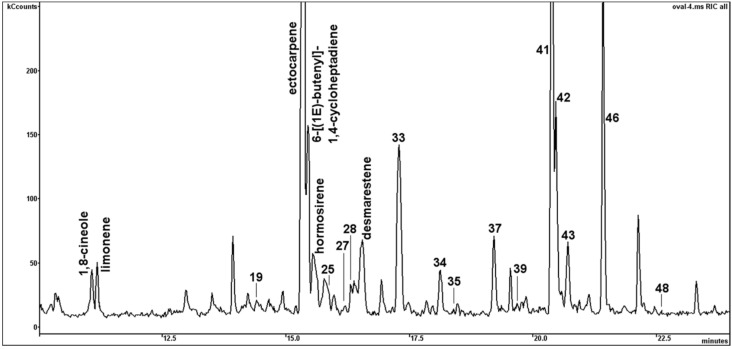
Example of GC-MS chromatogram showing the separation pattern of pheromonal VOCs and monoterpenes, detected in the BMB strain. Numbers indicate volatile compounds listed in Table 2.

The content of 6-((1*E*)-butenyl)-1,4-cycloheptadiene comprised 58.5% of the detected VOCs in the BMB strain. The gas chromatographic separation of pheromones of the BMB strain is depicted in Figure 5. These alicyclic olefins were totally absent in the volatile profiles of the CCAP strain.

**Table 2:** Volatile organic compounds (VOC) from BMB strain and CCAP strain of *Phaeodactylum tricornutum*, analysed by headspace SPME coupled with GC-MS. Structurally-identified compounds are marked in italics and characterized by their most prominent mass ion fragments (relative intensity in % in parentheses).

No.	RI ^#^	COMPOUND	BMB-E-0007; % of Total	CCAP 1052/1A; % of Total	MS Fragments
1	516	dimethyl sulfide	2.91	7.82	
2	746	dimethyl disulfide	0.42	-	
3	792	2-hexanone	-	2.31	
4	867	*p*-xylene	-	1,40	
5	941	α-pinene	0.06	0.84	
6	966	dimethyl trisulfide	0.25	-	
7	983	6-methyl-5-hepten-2-one	0.10	2.52	
8	1017	2,2,6-trimethyl-cyclohexanone	0.13	1.12	
9	1027	1,8-cineole	0.27	5.42	
10	1029	limonene	0.88	6.47	
11	1037	acetophenone	-	3.81	
12	1060	3-methyl decane	-	0.47	
13	1070	*aliphatic hydrocarbon*	0.11	5.68	*m/z* = 43, 57, 71, 85, 97, 111, 125
14	1077	*aliphatic hydrocarbon*	-	1.62	*m/z* = 43, 57, 71, 85, 98, 111, 123
15	1080	3-acetyl-2,5-dimethyl furan	0.17	-	
16	1099	nonanal	0.19	1.52	
17	1100	undecane	0.39	1.31	
18	1126	4-oxoisophorone	0.17	6.27	
19	1133	*putative pheromonal VOC*	1.12	-	*m/z* = 146(10), 117(100), 91(70), 104(45), 115(50)
20	1134	*aliphatic hydrocarbon*	-	1.99	*m/z* = 43, 57, 71, 85, 113
21	1141	*aromatic compound*	0.07	5.78	
22	1163	ectocarpene	5.41	-	
23	1166	6-((1*E*)-butenyl)-1,4-cycloheptadiene	58.52	-	
24	1169	hormosirene	0.21	-	
25	1176	*putative pheromonal VOC*	2.24	-	*m/z* = 148(10), 91(100), 79(50), 119(40), 105(35)
26	1179	*aromatic compound*	0.25	1.72	*m/z* = 122(100), 107(95), 77(78), 91(36)
27	1185	*putative pheromonal VOC*	0.63	-	*m/z* = 148(5), 91(100), 79(90), 105(45)
28	1187	*putative pheromonal VOC*	0.92	-	*m/z* = 146(45), 117(100), 91(90), 131(90), 115(75)
29	1190	octanoic acid	0.07	0.95	
30	1192	desmarestene	1.01	-	
31	1203	decanal	0.18	1.52	
32	1209	β-cyclocitral	0.06	1.57	
33	1213	*putative pheromonal VOC*	2.30	-	*m/z* = 148(50), 91(100), 105(60), 119(45)
34	1237	*putative pheromonal VOC*	0.34	-	*m/z* = 146(40), 91(100), 117(90), 131(40)
35	1246	*putative pheromonal VOC*	0.12	-	*m/z* = 148(45), 91(100), 79(50), 105(40), 119(40)
36	1249	*aromatic compound*	0.14	-	*m/z* = 148(25), 122(100), 107(85), 91(50), 77(45)
37	1273	*putative* *pheromonal VOC*	0.40	-	*m/z* = 148(50), 91(100), 105(50), 119(45), 77(40)
38	1284	nonanoic acid	0.15	3.78	
39	1291	*putative* *pheromonal VOC*	0.04	-	*m/z* = 162(17), 147(100), 119(60), 91(25)
40	1300	tridecane	0.10	4.99	
41	1310	*putative* *pheromonal VOC*	9.43	-	*m/z* = 162(3), 147(2), 91(100), 79(25), 105(22)
42	1313	*putative* *pheromonal VOC*	0.60	-	*m/z* = 148(7), 79(100), 91(70), 67(65), 135(25)
43	1322	*putative* *pheromonal VOC*	0.62	-	*m/z* = 146(15), 79(10), 91(80), 67(60), 53(40)
44	1327	*aliphatic hydrocarbon*	0.98	-	m/z = 43, 57, 71, 85
45	1335	cyclo-β-ionone	0.23	1.69	
46	1346	*putative* *pheromonal VOC*	2.24	-	*m/z* = 166(20), 67(100), 81(60), 95(55), 109(50)
47	1382	decanoic acid	0.04	1.37	
48	1388	*putative* *pheromonal VOC*	0.31	-	*m/z* = 164(30), 91(95), 79(70), 105(55)
49	1410	dodecanal	0.16	1.84	
50	1449	geranyl acetone	0.24	4.47	
51	1460	2,6-di-*tert*-butyl-*p*-benzoquinone	0.18	1.84	
52	1504	2,4-di-*tert*-butyl phenol	0.07	0.98	
53	1558	butyl decanoate	0.88	3.05	
54	1592	tetradecanal	0.10	1.38	
55	1595	dodecanoic acid	0.03	1.11	
56	1815	(*E*,*E*)-farnesyl acetate	0.16	1.43	
57	2000	eicosane	0.30	-	
58	2178	ethyl hexadecanoate	0.44	2.67	
59	2200	docosane	0.05	6.14	
60	2832	squalene	2.24	-	
61	3000	triacontane	0.34	1.13	
		sum %	100	100	
		Total MS detector response	1.56E + 07	1.28E + 06	

**^#^** Retention indices calculated based on a series of *n*-alkanes on an apolar DB-5 column.

## 3. Discussion

The interesting metabolic properties and the generally great phenotypic variation shown in *P. tricornutum* isolates motivated us to search for additional characteristics in growth, protein expression, FA profiles and VOCs in a BMB strain isolated from Western Norwegian fjord water. All experiments and analyses were run in parallel with a common CCAP strain for comparison. The BMB strain was shown to be dominated by oval cells (Figure 2A), and the CCAP strain (Figure 2B) by fusiform cells. The oval cell form of *P. tricornutum* has in several studies been emphasized to be a benthic life form, which is supported by the fact that fusiform cells grown on agar surfaces will transform into slightly motile oval cells surrounded by extracellular polymeric substances. It was also reported that oval cells transferred from agar into liquid medium would gradually revert to fusiform cells [19]. It is therefore an interesting feature of the BMB strain that it can be maintained oval in liquid bubbled cultures. Epigenetic changes have recently been shown to have an important role in phenotypic variation [23]. It is not unlikely that the polymorphism seen in *P. tricornutum* could be related to epigenetic phenomena. Epigenetic regulation has been shown for *P. tricornutum,* and extensive gene methylation correlated strongly with differential expression under specific conditions [24]. Proteomics can be one approach to reveal epigenetic effects [25], and here we have shown differences in soluble proteins separated by 2DGE between the BMB and CCAP strain in different growth phases. These differences indicate that the two *P. tricornutum* strains are under different transcriptional or epigenetic regulation, but a more in depth study is required to be able to reveal more about regulation of expressed proteins.

Growth parameters like cell numbers and dry weight and also expressed proteins are important parameters to describe physiological status of microalgae cultures. When the CCAP cultures had the maximal growth rates at day two, the highest number of expressed proteins on the 2DGE gels was observed (Figure 3D and Table 1). The successive increase in growth rate for the BMB strain from day one to day two and from day three to four co-occurred with an increased number of proteins on the 2DGE gels at day four (Figure 3B and Table 1). This is in accordance with the increased need for proteins in cell division when growth rates increase. A decreased number of separated proteins at day four is observed for the CCAP cells (Figure 3E), and this can indicate that a turnover of the proteins had occurred, a process that allows the cells to re-utilize amino acids and change protein content. These findings are supported by a recent study of *P. tricornutum* and its responses to nitrogen deprivation, where the authors showed that following nitrogen deprivation, a reduction in biosynthesis and an increase in recycling of N-containing compounds like amino acids, proteins and nucleic acid occurred at the transcript level [26]. It has also been shown that as the growth rate decreased, the overall rate of protein turnover decreased in *P. tricornutum* [27]. The highly reduced numbers of expressed proteins found in the early stationary phase (day 8) in our study have also been observed in a stationary culture of the dinoflagellate *Prorocentrum triestinum* [28]. Nitrogen limitation has been shown to decrease the cell protein content and abundance of proteins like ribulose bisphosphate carboxylase/oxygenase [29]. A characteristic protein observed for the BMB strain was one with molecular weight of about 33 kDa and *p*I of about 6.2 (Figure 3A–C). This finding did not correspond to the presence of a 21 kDa protein found in the fusiform cells detected from comparison of protein bands from clonal cultures of oval and fusiform cells in one-dimensional SDS-PAGE gels [30], hence the 33 kDa protein appears to be specific to the BMB strain.

Fatty acids are metabolites of high commercial interest and with many interesting cellular functions. In microalgae, they are building blocks in cellular lipids like the polar membrane lipids and the neutral lipids [31]. Some fatty acids have also been shown to possess antibacterial properties [32]. In our studies, the total amount of fatty acids per cell and per dry weight increased more than two times from exponential to stationary phase in both strains. At the same time, both strains showed a similar shift in their relative FA composition. The higher relative content of long chain polyunsaturated FA (especially EPA) in exponential phase changed to a higher fraction of monounsaturated and saturated fatty acids, especially palmitoleic acid (16:1 *n*-7) in the stationary phase. Both the increase in total FA and the shift towards saturation and monounsaturation in the stationary phase have been documented for *P. tricornutum* by others [33,34] and can be related to the accumulation of neutral storage lipids (triacylglycerols, TAG) as a response to nitrogen starvation in the stationary phase. They serve as storage compounds for carbon and energy and predominantly consist more of saturated and monounsaturated fatty acids, while polyunsaturated fatty acids (PUFA) are typically present in membrane lipids [31]. Interestingly, EPA has also been reported to have high antibacterial activity [35,36]. In another study, it was found that extracts from fusiform cells had higher antibacterial activity and higher content of the fatty acids EPA, hexadecatrienoic acid and palmitoleic acid than extracts from oval cells [32]. It is interesting to note that here in our study the BMB strain (dominated by oval cells) had higher relative amounts (Figure 4B) and cell specific amounts of palmitoleic acid (average 4.41 pg·cell^−1^ ) than the CCAP strain (average 3.50 pg·cell^−1^, dominated by fusiform cells) in the stationary phase.

Even if most of the VOCs described are highly volatile and hydrophobic, they are still slightly soluble in water and can be bioactive, *i.e.*, molecules can be sensed by other individuals of the same species or even other species in the context of defense.Volatile metabolites originating from exudates from living cells can give important information concerning physiological status, and several volatile compounds have been reported to be derivatives from degradation of fatty acids. More volatile metabolites could be detected from the oval cells (55) than from the fusiform cells (36). Interestingly the higher number of separated protein spots in the stationary phase of the BMB strain co-occurred with an increased number of detected VOCs. The bouquet of cultures of the BMB *P. tricornutum* strain has a distinct pleasant smell, probably from the high levels of 6-((1*E*)-butenyl)-1,4-cycloheptadiene or possibly from ectocarpene. The most striking findings of the VOCs analyses in our study were the relative high levels of ectocarpene, 6-((1*E*)-butenyl)-1,4-cycloheptadiene, hormosirene, and desmarestene and related structurally-assigned compounds, which were exclusively detected in the BMB cultures. The occurrence of ectocarpene in *P. tricornutum* and other diatom species have been reported earlier [37,38], and the production of hormosirene in the freshwater diatom *Gomphonema parvulum* has also been reported [7]. Ectocarpene, hormosirene and the intermediate 6-((1*E*)-butenyl)-1,4-cycloheptadiene have been identified from brown algae, where their biochemical function are described to be coupled to male gametophyte release and sexual attraction [39,40,41,42]. However, hormosirene has also been found in the brown algae of the genus *Dictyopteris* spp, showing no connection to sexual reproduction [43]. The occurrence of the pheromone dictyopterene C in *P. tricornutum* [37] could not be confirmed by our study. It is interesting to note that the pheromone desmarestene has not been detected in diatoms so far. The presence of C11 unsaturated olefins (pheromones) in the volatile fraction from cultures of the BMB strain is likely to originate from oxylipin chemistry. The biosynthesis of C11 unsaturated olefins in diatoms and brown algae is based upon eicosanoid precursors, and the cleavage of these fatty acids results in C11 hydrocarbons and oxoacids [7,10]. We have shown that the BMB strain contained high levels of potential FA precursors for bioactive oxylipins.

It is tempting to suggest that the presence of the pheromone “cocktail” released from the oval cells in the BMB strain reflects its benthic life stage where the oval cells are assumed to dominate, even if they were grown in liquid cultures in our study. To our current knowledge, the fusiform and oval cells, typically representing the CCAP and BMB strain, respectively, are not associated with sexual differentiation since comparison of the DNA content in three different cell types of *P. tricornutum* revealed no significant differences [44]. These pheromones and aldehydes may act as allelochemicals for the organisms in order to protect themselves against competitors or grazers, or it may be part of a quorum sensing strategy. Quorum sensing involves the production and detection of certain signalling molecules (also called auto-inducers and pheromones) by organisms [45]. The possible roles of these pheromones should be examined further.

Diatoms belong to the large group of Heterokontophyta, a major line of eukaryotic algae. Although there are phylogenetic differences between unicellular diatoms from the class Bacillariophyceae and the brown algae from the class Phaeophyceae, they share common biochemical features such as chlorophylls, carotenoids, fatty acids and hydrocarbons [37], and can thus, probably share pathways leading to the biosynthesis of pheromonal-active VOCs. This is also true with regard to the identification of terpenoid structures in our study, whose biosynthesis and abundance have high significance in terrestrial plants. Three monoterpenes were detected in our *P. tricornutum* strains, which are in accordance with the detection of terpenes such as α-pinene and limonene in diatoms and other phytoplankton species, recently being reported [46]. In addition to this, the oxygenated monoterpene 1,8-cineole and the triterpene squalene have been identified from a red algae species [47]. Another common feature is the release of S-containing volatiles (sulphides), which are known to be produced by both phytoplankton [48] and other marine organisms [49]. In contrast, halogenated VOCs [50] could not be detected by HS-SPME in our study probably due to high volatility and low abundance of these structures.

Many of the detected VOCs in our study (Table 2) are chemicals reported to exert antibiotic activity, such as 1,8-cineole [51], α-pinene and limonene [52,53] and octanoic acid (caprylic acid) [54]. Our findings indicate that *P. tricornutum* cells produce an arsenal of antimicrobial compounds probably to protect themselves against bacteria. Several studies have reported the transformation of polyunsaturated fatty acid (PUFA) into unsaturated aldehydes upon mechanical damage of diatom cells. These unsaturated aldehydes have been shown to have a negative effect on the copepod reproductive process, and they induce apoptosis in human carcinoma cells [55]. They are therefore thought to be a part of the defence system in diatoms thus contributing to an increase in the overall success of many diatom species [56]. Such unsaturated aldehydes were not detected in the VOCs from the strains in our study, but this is not unexpected since it depends upon mechanical breakage of cells, which typically occurs during grazing. However, water extracts from four isolates of *P. tricornutum*, including the BMB strain, strongly inhibited blood platelet activation and induced cell death in leukaemia cells. The origin of activity was not chemically identified, but it was shown not to originate from adenosine [21]. The apoptotic effects could come from oxylipins released when preparing the extracts, although further isolation and characterisation of the active compounds in water extracts must be conducted to confirm this suggestion. It is interesting to note that all of the four *P. tricornutum* isolates from Western Norwegian fjords were dominated by oval cells, and it is therefore tempting to suggest that cells representing a benthic life stage of *P. tricornutum* contain a variety of bioactive metabolites with the purpose of increasing the survival of cells living on surfaces in the intertidal zone.

We have presented data on growth and protein expression, which confirm that the BMB strain had the potential to fully exploit the growth conditions in our experiments and thereby obtain higher cell densities and dry-weights than the non-local strain, which is a prerequisite for biomass production of microalgae for aquaculture or even for biofuel [57]. The BMB strain also showed high levels of EPA, a known precursor of bioactive metabolites, often found in the volatile fraction of the culture. The results from the volatile profiling in our study suggest that further investigations of the diatom *P. tricornutum* should focus on asexual life stages both in pelagic and benthic environments, in order to reveal novel VOCs patterns and potential allelopathic functions in inter-species communication. Furthermore as it is realized that the life span for antibiotics is limited, there is a continuous search for new candidates [58]. Therefore, further investigations on the benthic diatoms as a source for new antibiotics, anti-cancer and other bioactivities should be intensified, adding new focus to other parts of the metabolome like the volatile organic compounds. The volatile fraction of large scale cultures of microalgae can be an important by-product that can be exploited and add value to the cultivation of microalgae. Local isolates of *P. tricornutum* like the BMB strain should be investigated for their biomedical potential, such as products of oxylipin chemistry, like 2,4-decadienal, which have been shown to induce apoptosis in cancer cells, and in copepods and sea urchin embryos [59]. Interestingly, many anti-cancer agents used by stalwart drugs share common properties with oxylipins derived from diatoms, including teratogenic and anti-mitotic activities [60].

In view of the wide distribution of diatoms, their role as the world’s most important primary producer and their metabolic capabilities, we strongly believe that increased knowledge of their biology in combination with improved analytical chemical methods will reveal many new and important metabolites in the years to come.

## 4. Experimental Section

### 4.1. Cultivation of BMB and CCAP Strains for Growth and Proteins Analyses

The BMB strain of *P. tricornutum* was isolated and maintained in stock culture as strain ND58 as described [21]. It is now an accession (BMB-E-0007) in Bergen Marine Biobank (BMB) at Department of Biology, University of Bergen, Norway and Uni Research AS. The second strain of *P. tricornutum* was strain CCAP 1052/1A from the Culture Collection of Algae and Protozoa in Oban, UK. Both isolates have been maintained under identical conditions since 1997 [22]. BMB and CCAP strain were grown as batch cultures at 20 °C in sterile Walne’s medium [61] prepared with artificial seawater (ASW) [62] with salinity of 27 PSU in 300 mL glass cylinders with 3.5 cm in inner diameter. Humidified air mixed with CO_2_ (1% CO_2_ final concentration) was filtered through bacterial filters (0.2 μm) and bubbled through the cultures. Cultures were provided with continuous white light of 249 µmol photons m^−2^·s^−1^ at the front of the cultures. Scalar irradiance was measured at the fronts of the cultures with a spherical sensor (Biospherical Instruments Inc. QSL-100, San Diego, CA, USA).

Six parallel cultures, each consisting of 280 mL, were grown as batch cultures. For every sampling for protein analyses, one of these cultures was harvested by centrifugation at 2000 rpm for 5 min in 50 mL aliquots at day 2, 4 and 8. The cells were carefully washed twice in sterile ASW before pelleted, frozen in liquid nitrogen and stored at −80 °C until analysed. The cultures were not axenic, but bacterial numbers were kept at a minimum using sterile technique, sterilized equipment and media. The presence of bacteria was observed daily in a microscope (100× magnification with immersion oil).

A Zeiss Axio Imager Z1 microscope (Carl Zeiss) was used to photograph representative cells from the two strains (100× magnification with immersion oil).

### 4.2. Growth Measurements

Cell counts, growth determinations and dry weight were performed as described in [22].

### 4.3. Preparation of Protein Samples

Each protein sample was prepared from three parallel extractions from three cell pellets, each from a culture volume of 50 mL. Each pellet was dissolved in 0.3 mL precipitation solution, consisting of acetone with 10% TCA (Trichloroacetic acid) and 20 mM DTT (Dithiotreitol, GE Healthcare Bio-Sciences, Pittsburgh, PA, USA). The cells were disrupted in the precipitation solution using glass beads (0.45 mm, BioSpec Products, Bartesville, OK, USA), and they were mixed with a vortex blender seven times for 1 min, allowing the samples to cool on ice between the pulses. The protein extracts were removed and the beads were washed twice with 0.5 mL of the precipitation solution, and these washes were pooled with the first extract and left to precipitate o/n at −20 °C. The precipitated proteins were centrifuged at 4 °C at 17,500 *g* for 30 min and the protein pellets were re solubilised in 1.5 mL acetone (with 20 mM DTT) to remove TCA and left to precipitate for 2 h at −20 °C. This was repeated at least twice or until the pellet appeared white with no remnants of pigments. The protein pellets were dissolved in 0.4 mL rehydration buffer containing 2 M thiourea, 7 M urea, 4% CHAPS, 0.5% Triton X-100, 20 mM DTT and 0.5% Zoom^®^ Carrier Ampholytes pH 3–10 or pH 4–7 from Invitrogen (Waltham, MA, USA) at room temperature overnight. Unsolved proteins were removed by centrifugation at 17,500 *g* at 4 °C for 30 min. Quantification of protein in each parallel extraction was performed using the Bio Rad RC DC protein assay (Bio-Rad, Hercules, CA, USA). The calibration curves were prepared using BSA dissolved in the same rehydration buffer as sample (0.2–1.5 mg/mL) and the absorption of the solution was recorded at 750 nm using a Shimadzu spectrophotometer (UV-2401PC). After the protein concentrations had been measured, the three parallel protein extracts were pooled, representing the sample from that time-point and strain.

### 4.4. Two Dimensional Gel Electrophoresis (2DGE)

ZOOM^®^ IPGRunner™ System from Invitrogen™ (USA) was used for the first dimension isoelectric focusing (IEF) and second dimension SDS-PAGE. The 7 cm immobilised pH gradient gels (IPG) Zoom^®^ Strips from Invitrogen™ pH 4–7 were used. Protein samples were applied to the strips as described by the manufacturer, and the best resolution in silver stained gels was obtained by loading 10 μg protein to each gel (*n* = 3).

IEF running conditions were modified according to the manufacturer’s recommendations and running conditions were as follows: 0.5 h at 100 V, 1 h at 200 V and 5 h at 500 V using the PowerEase^®^ 500 from Invitrogen. After IEF the strips were frozen as recommended [63] overnight at −20 °C before equilibrated in DTT and IAA (Iodoacetamide, Sigma Aldrich, St. Louis, MO, USA) before running the second dimension as described in the manual. The second dimension was performed using NuPage^®^ Novex 4%–12% Bis-Tris Zoom^®^ Gels from Invitrogen and comigrated with a broad range molecular weight marker (2.5 to 200 kDa). SDS-PAGE was run for 50–60 min at 200 V.

### 4.5. Visualization, Imaging and Analysis of Gels

The gels were stained with a MS compatible SilverQuest™ Silver Staining Kit from Invitrogen (cat. no. LC6070). Gels were scanned using Bio imaging system from SynGene, and images were saved in TIFF format before analysed with Delta 2D version 3.5 (DECODON). Images were filtered to remove the background noise, and gel pictures of three parallel gels from the same sample preparation were fused to create a master gel representative for that particular sample. Another master gel was prepared from three parallel gel pictures from the other sample preparation, and these two master gels were again compared to monitor the reproducibility of the sample preparation procedure and the 2DGE run. The master gels were compared, day 2 with day 4, day 4 with day 8 for BMB strain and the same for the CCAP strain. Next, day 2 for oval cells were compared with day 2 for fusiform and so on and analysed for differences in the number of expressed protein spots (Figure 3).

### 4.6. Cultivation of Strains for Determination of Total Fatty Acids and Profiling

Two parallel batch cultures of the BMB (BMB-E-0007) and the CCAP strain were grown in sterile Walne’s medium as previous, but prepared with aged seawater and distilled water (80:20, v:v, 29 PSU) in 300 mL glass cylinders as described in Section 4.1, using a light intensity of 150 µmol photons m^−2^·s^−1^. Cultures were sampled daily for cell counting, pH, and once during mid exponential (day 3) and late stationary phase (day 10) for fatty acid and dry weight analysis. Counts of algae cells were performed by flowcytometry (FCM, FACSCalibur, Becton and Dickinson, Franklin Lakes, NJ, USA) for 60 s at high flow velocity with cultures diluted to give 20–100 events/s. The exact flow rate R (µL·min^−1^) was calculated by applying culture medium to the FCM and using the following equation (W_i_ − W_f_)/*t*·*ρ*, with W_i_ = initial weight (mg), W_f_ = final weight (mg), *t* = time (min), *ρ* = density (1 g·cm^−3^). Dry weight concentration was determined using 0.5 M ammonium formate as washing buffer as described [64]. For fatty acids determination, 10 mL of microalgal culture were sampled in 10 mL glass tubes (PYREX), centrifuged for 6 min at 4000 *g* and relived from the supernatant. Pellets were stored in nitrogen atmosphere at −20 °C until analysed.

### 4.7. Fatty Acid Extraction

Samples were derivatized to fatty acid methyl esters (FAME) by direct esterification according to [65]. Briefly, the sample pellet was dried in the 10 mL tubes by evaporating water under nitrogen atmosphere. Internal standard, 23:0 FAME dissolved in isooctane, was added (150 μL, 0.240 mg/mL or 100 μL, 4.90 mg/mL for samples in exponential or stationary phase, respectively). After evaporating the solvent, 0.5 mL methylation reagent (2 M HCl in methanol) was immediately added to each tube. The tubes were flushed with nitrogen, sealed and incubated in oven at 90 °C for 2 h. After cooling to room temperature, half of the methanol was evaporated and 0.5 mL water was added. The samples were thereafter extracted twice by 1 mL isooctane. Before analysis by gas chromatography (GC), the combined extracts were further diluted by isooctane (1:2 or 1:20 for exponential or stationary phase samples, respectively).

### 4.8. FAME Analysis by Gas Chromatography and Mass Spectrometry (GC-MS)

The FAMEs were analysed on a 7890 gas chromatograph (Agilent, Santa Clara, CA, USA) equipped with autosampler, split-splitless injector, flame ionization detector (FID) and a 60 m BPX70 capillary column (SGE, Ringwood, Australia) with internal diameter 0.25 mm and film thickness of 0.25 µm. Samples of 1 µL were injected splitless at 60 °C where the temperature was held for 3 min before it was raised by 40 °C/min to 150 °C followed by 1.5 °C/min to 230 °C. Helium was used as carrier gas in constant flow mode with an estimated average velocity at injection of 30 cm/s. Injector and detector temperatures were 250 °C and 300 °C, respectively.

Identification of FAMEs was performed by analyzing selected samples on an Agilent 7890/5977 GC-MS system, using the same capillary column as in GC-FID. Conditions and methodology were as described in [66]. Data handling of GC-FID and GC-MS data were performed in Chrombox C and Chrombox Q, respectively (www.chrombox.org).

### 4.9. Sampling of Volatiles by Headspace Solid-Phase Microextraction (SPME)

Preliminary tests with non-polar and semi-polar SPME fibre types showed that the 75 µm Carboxen™/PDMS fibre (Supelco Inc., Bellefonte, PA, USA) showed highest sensitivity towards the broad range of structurally different volatile organic compounds (VOCs) from cultures of *P. tricornutum.* The fibre, mounted on a manual SPME holder (Supelco Inc.), was exposed to the GC inlet in a blank run for 2 min for thermal desorption at 220 °C prior to headspace sampling. 2.5 mL of each *Phaeodactylum* strain in early stationary phase (*n* = 3) was sealed in a 15 mL screw-capped vial with a phenolic cap and a PTFE/silicone septum (Supelco Inc.). The SPME fibre was exposed to the sample for 7 h by manually penetrating the septum (0.25 cm depth). The SPME device was immediately inserted into the GC injector and the fibre thermally desorbed for 3 min at 220 °C.

### 4.10. Volatile Analysis by Gas Chromatography and Mass Spectrometry (GC-MS)

A Varian Star 3400 CX gas chromatograph (Varian Inc., Walnut Creek, CA, USA) coupled with a Varian Saturn 3 mass spectrometer were used for all analyses. An Agilent J & W DB-5 fused silica capillary column was applied for the chromatographic separation of VOCs: 30 m × 0.25 mm i.d., 0.25 μm film thickness. Helium was used as carrier gas (15 psi) at 50 mL/min through the injector and 30 cm/s through the column. The injector temperature was set at 220 °C carried out in splitless mode for 2 min. The GC temperature was a held at 40 °C for 2 min, ramped from 40 °C to 220 °C at a rate of 4.5 °C/min, and finally held at 220 °C for 3 min. The MS detector was set at 220 °C and a mass range of *m/z* 25–300 was recorded. All mass spectra were acquired with electron impact ionization. VOCs were tentatively identified based on mass spectrum database search (NIST/EPA/NIH mass spectral library v.2.0, 2005), in combination with retention indices and comparison of mass spectra found in literature. Based on compound-characteristic MS fragmentation patterns, several VOCs were structurally-annotated (Table 2) relating to the groups of aliphatic hydrocarbons, aromatic compounds or pheromonal VOC.
